# Globular Proteins and Where to Find Them within a Polymer Brush—A Case Study

**DOI:** 10.3390/polym15102407

**Published:** 2023-05-22

**Authors:** Aikaterini A. Galata, Martin Kröger

**Affiliations:** Magnetism and Interface Physics, Department of Materials, ETH Zurich, CH-8093 Zurich, Switzerland; mk@mat.ethz.ch

**Keywords:** linear polymer brushes, hydrophobic, polar, protein, interaction, ligands, surface, adsorption, potential of mean force

## Abstract

Protein adsorption by polymerized surfaces is an interdisciplinary topic that has been approached in many ways, leading to a plethora of theoretical, numerical and experimental insight. There is a wide variety of models trying to accurately capture the essence of adsorption and its effect on the conformations of proteins and polymers. However, atomistic simulations are case-specific and computationally demanding. Here, we explore universal aspects of the dynamics of protein adsorption through a coarse-grained (CG) model, that allows us to explore the effects of various design parameters. To this end, we adopt the hydrophobic-polar (HP) model for proteins, place them uniformly at the upper bound of a CG polymer brush whose multibead-spring chains are tethered to a solid implicit wall. We find that the most crucial factor affecting the adsorption efficiency appears to be the polymer grafting density, while the size of the protein and its hydrophobicity ratio come also into play. We discuss the roles of ligands and attractive tethering surfaces to the primary adsorption as well as secondary and ternary adsorption in the presence of attractive (towards the hydrophilic part of the protein) beads along varying spots of the backbone of the polymer chains. The percentage and rate of adsorption, density profiles and the shapes of the proteins, alongside with the respective potential of mean force are recorded to compare the various scenarios during protein adsorption.

## 1. Introduction

Proteins were, are and will remain a significant topic to be studied as they are necessary for the existence of life. Numerous studies exist, in the form of either experiments, scaling theory, or simulations, that extensively look into the overall behavior of individual proteins to characterize and understand their behavior, as this is not always an easy task. Proteins are not only large molecules imposing a large intrinsic number of degrees of freedom and therefore a huge number of possible conformations, but also interact with their environment [1,2,3,4,5]. Examples are confining surfaces or surrounding molecules adding to the number of preferred conformations they might explore. Despite their uncountable number of possible configurations, proteins fold spontaneously within milliseconds or seconds based on existing local interactions until they reach a folded metastable state of local minimum energy, according to the Levinthal paradox [6,7].

Proteins come in different sizes and shapes; such as α-helices, β-sheets and globular structures [8,9,10,11,12,13]. The latter ones are the proteins of interest for this study. Since each protein has its own 3D structure, the folded state is unique for each protein. A vast number of atomistic [14,15,16,17,18,19,20,21,22,23,24,25] and coarse-grained (CG) simulations [26,27,28,29,30,31,32,33,34,35,36,37,38,39,40,41,42,43,44,45,46,47,48,49,50,51,52,53,54,55] studied the conformational dynamics and other properties of proteins. While atomistic simulations are often preferred due to their accuracy and underlying chemical information, the computational cost to reproduce a trustworthy result is an important drawback, and makes them prohibitive to use in many cases, including adsorption phenomena of explicit proteins by polymer brushes. Therefore there is a trade-off of accuracy for time by the use of CG models, which often preserve the main microscopic characteristics upon ignoring many of the constraints, and the degrees of freedom. In the case of proteins such characteristics could be the effect of excluded volume or sequence-dependent intrachain interactions, while in the case of polymer brushes, which will constitute the adsorption surface in this study, it could be the overall conformation and location of the proteins and polymers. As far as it concerns the CG simulations of proteins there are several models including lattice Monte Carlo (MC) [41,42,43,44,46,47,56,57] and continuum Molecular Dynamics (MD) [45,48] simulation models.

Our interest in the adsorption of globular proteins led us to choose for this study a variation of the well-known CG hydrophobic-polar (HP) protein folding model, proposed by Dill and Chan in 1985 [58]. According to this model, protein folding is based on amino acid sequences and their mutual interactions due to their hydrophobic or polar nature, respectively. In other words, hydrophobic amino acids are attracted to one another and avoiding water molecules at the protein’s outer surface, as it is more favorable energetically. The hydrophobic amino acids then get accumulated in the center of globular proteins, surrounded by hydrophilic/polar ones. It has been shown that the stronger the hydrophobic interactions, the more stable the protein structure [59]. Proteins simulated by the HP model are known to exhibit diverse conformations that lie close to the global minimum conformation due to the similar energies of their metastable energy states (the same applies when they are in the vicinity to a surface) [58,59]. These differing conformations exhibit geometrical similarity; for this reason they are close to one another on the energy landscape. These conformations are accessed during a folding procedure, where the protein looks for conformations that will lead to an energy decrease, while Brownian motion is still causing the adoption of an ensemble of conformations [58].

Polymer surfaces, upon which we study the behavior of globular proteins, are known to be mechanically stable (and in some cases biocompatible), to have low cost and wide applicability which adds to our choice for the surface substrate [60,61,62,63]. Polymer brushes, often made up by grafting polymer chains by one end to a surface, are the ones that usually constitute polymer surfaces, and their applications have gained a lot of attention [62,63,64,65,66,67,68,69,70,71,72]. In contrast to the wide range of studies for proteins or polymer brushes, a combined study is much less common. The existing studies have been carried out for polystyrene, polyethylene and polydimethylsiloxane [25,73,74,75,76] and there are also a few CG MC studies [57,77], while we choose to explore the adsorption and conformational dynamics of the polymer+protein CG model via MD.

Within the present study we do not focus on a particular protein species and polymeric surface. Instead, we investigate a generic CG model that is characterized by the strength of tethering surface attraction, size of proteins, polymerization degree of polymers, location of ligands etc. to be able to develop an understanding on the effect of these characteristics on the dynamics and properties of the protein-brush complex. The model, its parameters, and the methods are presented in Section 2, followed by an analysis and discussion of the effect of parameters on the adsorption properties of proteins in Section 3. Conclusions are provided in Section 4. A number of additional results are provided in appendices.

## 2. Model and Methods

### 2.1. Coarse-Grained Model and Simulation Setup

The system under study consists of a planar polymer brush, water, and proteins, using a bead-spring representation. The polymer brush is made from *G* flexible and linear polymer chains, each made of *N* beads (repulsive R- or attractive A-beads), and tethered by one end to an implicit wall at z=0. Each protein is formed by a linear, but heterogeneous polymer chain consisting of $N_{p}$ beads that interact with one another and are either hydrophilic (P-beads) or hydrophobic (H-beads). Water is modeled by monomeric w-beads. Interactions between bead types and the bead coloring scheme are summarized in Table 1. In the current section we elaborate on the details of each part of our system as well as the parameters to be varied lateron (as for example, the implicit wall attraction towards the proteins or the size of the proteins) in order to study their behavior close to a polymeric surface.

If not otherwise mentioned, we are going to use reduced, dimensionless LJ units throughout, so that results apply to arbitrary choices of their dimensional counterparts. Unit mass is the mass of a bead, unit length is the distance between two neighboring beads beyond which they do not repel each other (an effective particle diameter), and unit energy is $k_{B}T$. Every dimensionless number mentioned in the following can thus be converted to a dimensional value, if its physical units are known, and if unit mass, length, and energy have been specified.

All results to be presented in this study have been obtained using a 54.29×64.44×20 simulation box with a fixed size and shape, and fixed total number of 45510 beads. The box thus exhibits a rectangular area A=3498.4 serving as tethering *x*-*y*-plane (surface, and wall), and height H=20 in *z*-direction. The resulting constant bead number density is n=0.65, reminiscent of dense liquid. Periodic boundary conditions apply in the lateral *x*- and *y*-directions.

#### 2.1.1. Brush Setup

Planar polymer brushes at different surface grafting densities σ (chains per surface area *A*) are created in the presence of water. To this end, the linear flexible CG chains that consist the brush are permanently tethered by one of their terminal beads to the *x*-*y*-plane at altitude z=0. The tethered surface beads are assumed to be immobile, and the system is periodic only in the *x*- and *y*-directions. *G* grafting points (i.e., tethered surface beads) are distributed uniformly on the surface of total area *A*; thus, the surface number or grafting density of polymer chains is σ=G/A. The interaction between all (except the tethered) beads and an implicit wall at z=0 is governed by a 9-3 potential

(1) $${}_{\epsilon }W_{z_{c}}\left(z\right)=\left.\left\{\begin{array}{cc} \epsilon \left(\frac{2}{15}z^{-9}-z^{-3}-\frac{2}{15}r_{c}^{-9}+z_{c}^{-3}\right), & z\leq z_{c}\\ 0, & z>z_{c} \end{array}\right.\right.,$$

parameterized by a dimensionless energy depth ϵ and cutoff distance $z_{c}$, and characterized by interaction strength *I* (Table 2). There are two implicit planar and parallel walls in the system; one at z=0 (tethering surface), another at z=20, using the shifted ${}_{\epsilon }W_{z_{c}}\left(20-z\right)$. All non-tethered beads that belong to the polymer and water are repulsed by the wall at z=0 (the cut off distance is taken in the minimum of the potential, i.e., at $z_{c}={\left(2/5\right)}^{1/6}\approx 0.858$), while protein beads are attracted by the tethering surface as a whole (both its hydrophobic and polar parts, more details in Section 2.1.2). On the contrary, the wall at z=20 repulses all beads (brush, water and protein) with $z_{c}\approx 0.858$.

Each polymer chain consists of N=50 beads that are permanently joined together via a bond potential. All beads (tethered and non-tethered) are assumed to have identical masses and effective diameters, mediated through the repulsive part of the LJ potential,

(2) $${}_{\epsilon }LJ_{r_{c}}\left(r\right)=\left.\left\{\begin{array}{cc} 4\epsilon \left(r^{-12}-r^{-6}-r_{c}^{-12}+r_{c}^{-6}\right), & r\leq r_{c}\\ 0, & r>r_{c} \end{array}\right.\right.$$

also parameterized by a dimensionless energy depth ϵ and cutoff radius $r_{c}$. For the special choice of ϵ=1 and $r_{c}=2^{1/6}$ the ${}_{\epsilon }V_{r_{c}}\left(r\right)$ is known as Weeks-Chandler-Anderson (WCA) potential [78].

For the intramolecular bonded interactions between polymer beads we used the classical finitely extendable nonlinear elastic (FENE) bonds residing between each two consecutive CG beads along the polymer backbone via the use of the FENE potential [79,80,81,82], that relatively poorly approximates the inverse Langevin function [83]. This potential is given as function of the separation *r* between adjacent (“chemically” bonded) bead centers as

(3) $$V_{\mathrm{FENE}}\left(r\right)=-\frac{k_{\mathrm{FENE}}R_{\mathrm{FENE}}^{2}}{2}\ln\left[1-{\left(\frac{r}{R_{\mathrm{FENE}}}\right)}^{2}\right]$$

where $R_{\mathrm{FENE}}$ is the maximum spatial separation between FENE-bonded beads within the polymeric chain, and $k_{\mathrm{FENE}}$ is a spring coefficient. In our systems the values for the FENE constants are chosen as $k_{\mathrm{FENE}}=30$ and $R_{\mathrm{FENE}}=1.5$, and the temperature is set to T=1, following previous studies of polymeric systems [79,81,84,85]. The polymer chain that is tethered by one end to the implicit wall has initially a rodlike conformation. Water is added randomly without overlap (i.e., the minimum distance between each pair of beads is above unity at startup) to the system before its equilibration. Each water molecule is represented by a CG bead that interacts with all beads via the WCA potential.

Molecular dynamics simulations are carried out using Large-scale Atomic/Molecular Massively Parallel Simulator (LAMMPS) [86] under NVT conditions, at a constant bead number density n=0.65. The integration time step chosen is Δt=0.005 and the temperature is controlled via a Nosé-Hoover thermostat with a temperature damping parameter (a time) of 1 for a duration of at least $t=2.5\times 10^{6}$ ($10^{8}$ steps), depending on each individual system.

The grafting densities that are studied correspond both to the mushroom/intermediate and the brush regime. Since the polymer brush consists of flexible chains, as brush regime we define the high density regime, where the radius of gyration of the polymer, $R_{g}$, exceeds the mean distance, which is roughly equal to $1/\sqrt{\sigma }$, between neighboring grafting points [70], or equivalently, where the squared gyration radius exceeds the mean surface area per chain, $\Sigma =\sigma^{-1}=A/G$. In any other case, one is either at the mushroom regime, where there is no interaction among the chains grafted to the surface because $R_{g}^{2}\ll \Sigma$, or at the intermediate regime for $R_{g}^{2}\approx \Sigma$. Therefore, for the remainder of this manuscript, the system with grafting density σ=0.023 is going to be called ’mushroom’, the one with σ=0.056 ’intermediate’ and the one with σ=0.087 ’brush’. To realize these grafting densities at unchanged grafting area *A*, we varied the number of polymer chains, G=81, 196 and 306, respectively. From now on, when referring to the chains tethered to the implicit wall irrespective of their grafting density, we will mention them as ’coating’.

#### 2.1.2. Protein Setup

**Figure 1:**
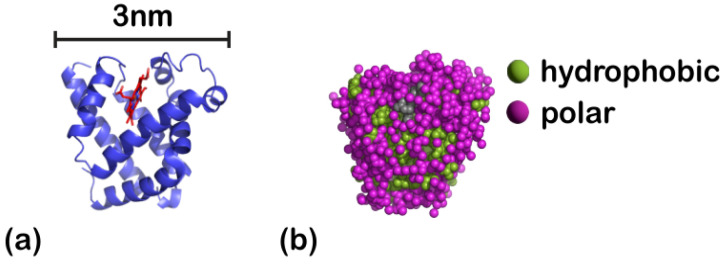
(**a**) Ribbon diagram and (**b**) coarse-grained representation of myoglobin at room temperature in water, in which each aminoacid is represented by one sphere. In total, in this myoglobin representation, there are 151 aminoacids kgalong with two sulfate ions and a core made of protoporphyrin IX containing Fe (also called HEM). Each of the aminoacids has roughly a mass equal to 110 Da and average volume of about 139 Å${}^{3}$ [93]. The diameter of the myoglobin protein is ≈3 nm [94]. From the volume per aminoacid and the hydrophobicity by the sequence, the fraction of its hydrophobic beads is approximately 40% [87,88,95], In (**b**) the purple spheres stand for the polar aminoacids, while the green ones for the non-polar ones, and grey ones for the sulfate ions and HEM. The CG model we are employing here uses a generic bead-spring representation of proteins such as myoglobin, where each bead (either hydrophobic or polar) represents one or more aminoacids.

An off lattice hydrophobic (H)-polar (P) model is adopted to obtain the native structure of the proteins. In the HP model, the amino acids are classified into H- and P-beads. [50,52,58] This doesn’t mean necessarily that each of these H- and P-beads has to represent a single aminoacid; depending on the coarse-graining while keeping in mind that any of the hydrophobic or polar beads might in reality represent one or more amino acids, depending on the choice for the coarse-graining. As an example, in Figure 1, we show a possible coarse-grained representation of the protein myoglobin [87] (data taken for deoxy-myoglobin with entry authors Vojtechovsky et al. [88]), for which each bead represents one aminoacid. The purple beads, that mostly occupy positions on the outer part of the protein, are the polar aminoacids, while the green ones, that are mostly placed on the inner part of the protein, stand for the hydrophobic/non-polar aminoacids [89,90,91]. The illustration of myoglobin was produced through PyMOL [92].

**Figure 2:**
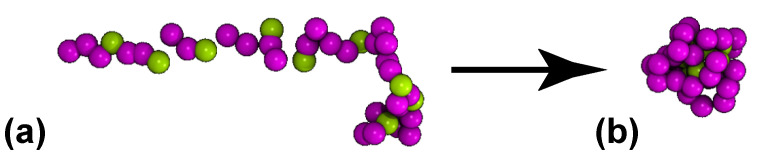
Individual CG protein S${}_{25}$ used in the present study, with $N_{p}=40$ beads and hp =25%, dissolved in CG water. Starting from an almost rodlike-shaped conformation at (**a**) t=0, the protein collapses at (**b**) t=250 and the hydrophobic H beads (green) are placed on the inner parts of the protein. The purple beads represent the polar P-beads.

Comparing this CG representation to our own in Figure 2, one can notice that the tendency of the non-polar beads to stay away from the water (as observed in Figure 1) is accurately reproduced by our model. Still, it is important to pinpoint that the HP-model has known deficiencies; although it reproduces structures that resemble real systems, it cannot capture important aspects of it. For example, all of the hydrophobic residues have a strong preference to stay to the inner part of the protein, while all of the polar ones prefer to interact with water. This might serve as a good approximation on a conceptual level, but in reality a high percentage of the exposed surface in the native state of globular proteins is nonpolar and hydrophobicity is not the only governing factor defining the structure of the protein [96,97,98]. In the present study each of the proteins has a different random sequence of HP-beads to showcase that the behavior is qualitatively universal for the same ratio of hydrophobic to polar beads (Figure 2). Two ratios of hydrophobic to polar beads were studied in order to understand how this ratio affects the behavior of the protein close to the coating; one hydrophobicity ratio, hp, equal to 25% and one equal to 35%.

As far as it concerns the size of the protein, there are two cases studied. In the first case, each short (S) protein consists of a single chain of $N_{p}=40$ beads (25% or 35% of them are hydrophobic and the remaining ones hydrophilic) and in the second one, each long (L) protein consists of a single chain of $N_{p}=60$ beads (who are again 25% or 35% hydrophobic). These proteins are denoted as $S_{25}$, $S_{35}$ and $L_{25}$, $L_{35}$, respectively. Since protein folding is mainly governed by the relative hydrophobic character of the amino acids, an attractive ${}_{120}$LJ${}_{1.19}$ interaction is set for the hydrophobic interactions following Equation (2), while all beads of the protein interact completely repulsively via the WCA potential with the rest of the beads that consist the system (i.e., the polymer coating, the water and the hydrophilic beads of the proteins). The bonds of the protein beads are given by Equation (3), having $k_{\mathrm{FENE}}=30$ and $R_{\mathrm{FENE}}=1.5$ as for the polymers.

**Figure 3:**
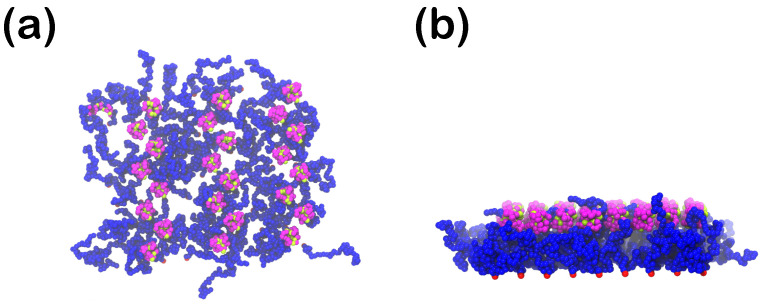
Start configuration (t=0) of the planar polymer mushroom (grafting density σ=0.023) in the presence of 24 S${}_{25}$ proteins. The water beads are not shown for clarity. (**a**) top view from the positive *z*-direction, perpendicular to the surface, (**b**) side view. Color scheme for beads according to Table 1: R (blue), H (green), P (purple), anchored immobile R beads are red.

Molecular dynamics simulations are carried out using the LAMMPS software [86] under NVT conditions for a single protein for each system size in an CG aqueous environment (where water consists of repulsive monomeric beads obeying the WCA potential), at a constant number density n=0.65 at integration time step Δt=0.005 up to t=5000 ($10^{6}$ steps), starting from a rodlike conformation for each protein size. The temperature is controlled once again via a Nosé-Hoover thermostat with the same damping parameter as for the coating. In the course of these single-chain simulations, we retrieve $G_{p}=24$ protein conformations and place them close to the upper part of the polymer coating. The value for $G_{p}$ was chosen so that the proteins can be distributed on the coating, without them causing crowding on the upper bound of the coating (Figure 3). The implicit wall at z=0 is always attractive towards the proteins, with the attraction strengths varying from slightly attractive to highly attractive, i.e., $\epsilon_{\mathrm{wall}}=1$ or 3 for $r_{c,\mathrm{wall}}\in \left\{1.0,1.5,2.0,2.5\right\}$.

**Figure 4:**
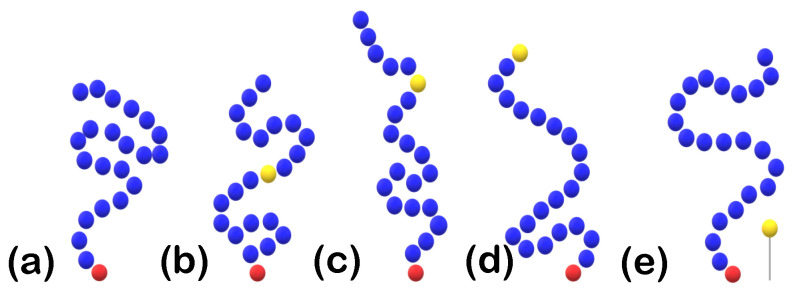
Schematic drawing. Placement of attractive (${}_{3}$LJ${}_{1.5}$) A-beads (yellow) considered in this study. For a tethered polymer chain (blue non-tethered, red tethered), we see (**a**) an un-doped chain, (**b**) A-beads at chain’s center, (**c**) A-beads at a random position, (**d**) A-beads at the terminal position, and (**e**) A-beads as ligands tethered at fixed altitude (z=3) over the surface.

In the following subsections, we study various cases for the polymer coating-proteins system (Figure 4), starting from a coating that contains an attractive backbone A-bead towards the hydrophilic part of the proteins for two possible locations: the attractive bead being the middle bead of each chain and the attractive bead being at a random position within each chain of the coating. Next we study the protein behavior when this attractive bead lies at the free end of each chain of the polymer coating. Finally, as a last case study, we place extra CG beads that are again attractive towards the hydrophilic part of the chain representing ligands for two ligand densities at z=3. The aforementioned coating systems that are changed for the various case studies are now to be described in detail. (The proteins remain unaltered.)

#### 2.1.3. Coating Setup with Backbone Attractive Beads

The coating consisting of chains of N=50 beads for the initial system has no attractive beads towards the protein; in other words, it consists of two CG bead types, one representing the beads of the main chain and one representing the beads tethered to the implicit wall (Figure 4a). All coating systems, but for the ones containing ligands, will have one extra atom type (A-bead) at their backbone representing the attractive part of the polymer coating towards the hydrophilic part (P-beads) of the protein. For this type of alterations, no beads are added, just the atom type of specific beads is changed. For the coating setup with attractive backbone beads one bead-type is changed in each and every chain. There were two options studied for this case: 1. the middle bead of the polymer chain was chosen to be attractive towards the polar part of the proteins (Figure 4b) and 2. a CG bead at a random position within the chain was chosen to be attractive (Figure 4c). The coating setup remains as described. We study two polymer-protein attraction strengths, ϵ∈{1,3}, for $r_{c}=1.5$.

#### 2.1.4. Coating Setup with Terminal Attractive Beads

With a similar mindset as the one described in Section 2.1.3, we set the interactions of the free end of each polymer chain towards the hydrophilic part of the protein to be attractive, i.e., replace each terminal polymer R bead by an A-bead (Figure 4d). This attraction is once again given by Equation (2) for ϵ∈{1,3} and $r_{c}=1.5$, i.e., interaction potential ${}_{\left\{1,3\right\}}$LJ${}_{1.5}$.

#### 2.1.5. Coating Setup with Ligands

**Figure 5:**
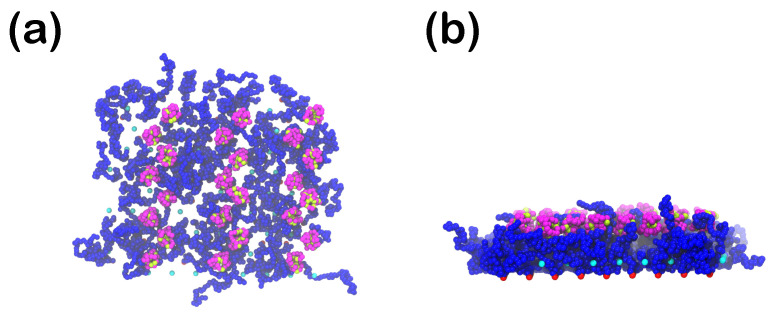
Same as Figure 3, now with additional ligands at z=3 with attraction strength ${}_{1}$LJ${}_{1.5}$, at ligand surface density $\sigma_{\mathrm{lig}}=0.023$, for the polymer mushroom in the presence of S${}_{25}$ proteins. Unchanged coloring scheme. (**a**) top view (**b**) side view.

For the incorporation of ligands into the system we add extra CG beads to the expense of water beads (Figure 4e and Figure 5, where ligand A-beads are depicted with a light blue color), while the coating and the proteins remain unchanged. These extra beads are kept at a fixed position at z=3 throughout the simulation, where at z=0 we have the implicit wall that is attractive towards the proteins. The ligands have a uniform distribution in the *x*-*y*-plane with surface density (ligands per area) equal to $\sigma_{\mathrm{lig}}=0.023$ (high density ligands) or $\sigma_{\mathrm{lig}}=0.01$ (low density ligands). They interact with the hydrophilic part of the protein through either the weakly attractive ${}_{1}$LJ${}_{1.5}$ or the strongly attractive ${}_{3}$LJ${}_{1.5}$, cf., Equation (2).

## 3. Results and Discussion

There are multiple reasons leading to protein adsorption on a polymeric surface. Therefore, in an effort to group them, we classify adsorption in three main categories: (i) primary adsorption, caused by the attraction of proteins by a bare, solid surface (implicit wall at z=0), (ii) secondary adsorption at the outer surface of the coating in order to avoid the free energy penalty caused by the insertion of proteins into the coating (which affects both the equilibrium adsorption and the rate of adsorption), and (iii) ternary adsorption within the coating itself due to monomer-protein attraction (where A-beads, either mobile or immobile, are the monomers that attract the proteins.) [53,99,100].

In this study one can see all types of adsorption taking place, as will be shown in the following subsections. To that end, we record the rate and the percentage of adsorption (Section 3.1) as well as the protein density profiles (Section 3.2) for the cases mentioned in Section 2, so that we get a better understanding of which parameters crucially affect protein adsorption (either by speeding it up or slowing it down, or even by prohibiting it) and which can be better used in potential experiments to tune the wanted result. In Section 3.3, we showcase what the shape of the proteins is for indicative case studies by studying their asphericity values. In the end, the Potential of Mean Force (PMF) among proteins and the coating due to adsorption is studied in Section 3.4.

### 3.1. Rate and Percentage of Protein Adsorption

#### 3.1.1. Effect of the Grafting Density

One of the most significant parameters affecting the adsorption of proteins on a polymer coating is the grafting density of the chains consisting the coating. Even though other factors might come into play, the density of the polymer coating itself is indicative of the freedom given to any protein to move within the coating, keeping in mind it sets a barrier related to the excluded volume and the free energy penalty that needs to be overcome for the proteins to get adsorbed. In this study there were three grafting densities studied going from the mushroom to the brush regime: σ=0.023 (mushroom), 0.056 (intermediate) and 0.087 (brush).

To grasp the size of the polymer chain of the coating and the one of the protein, we calculate the radius of gyration, $R_{g}$, and the end-to-end vector, *R* of the respective chains averaged for the last 30% of the data of each simulation. The squared radius of gyration, $R_{g}$, of an individual polymer or protein chain is computed as

(4) $$R_{g}^{2}=\frac{1}{N}\sum_{i=1}^{N}{\left(r_{i}-r_{\mathrm{cm}}\right)}^{2}$$

and the squared end-to-end distance, $R^{2}$, as

(5) $$R^{2}={\left(r_{N}-r_{1}\right)}^{2},$$

where $r_{i}$ denotes the position of the *i*th monomer and $r_{\mathrm{cm}}=N^{-1}\sum_{i}r_{i}$ the center of mass of this chain. In addition to the aforementioned quantities, we calculate the height *h* of each polymer chain as

(6) $$h=\max\{z_{i}\},\;i=1,...,N$$

where z(i) is the altitude of its *i*th bead with respect to the tethering surface. The time- and chain-averaged $R_{g}\equiv {\langle R_{g}^{2}\rangle }^{1/2}$, $R\equiv \langle R^{2}\rangle$ and $h_{\max}\equiv \langle h\rangle$ retrieved from these calculations are shown for the case of an equilibrated pure coating in the absence of proteins in Table 3. The corresponding conformational properties $R_{g,p}$ and $R_{p}$ for the protein chains in water, but in the absence of the coating are collected in Table 4.

**Figure 6:**
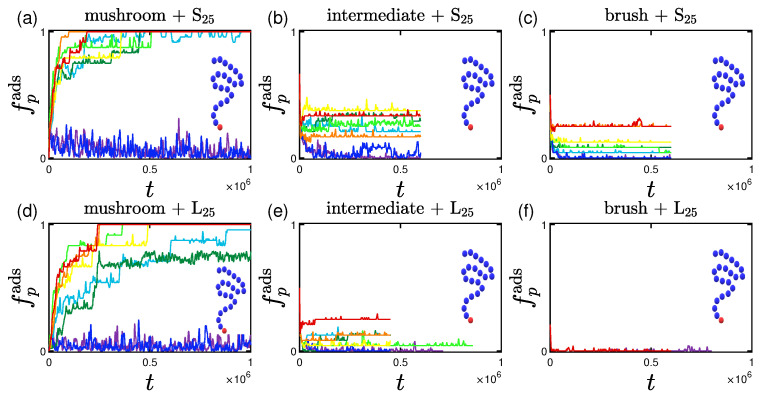
Effect of coating type (horizontal), protein size (vertical) for different attractive wall strengths (color code defined in Table 2). Rate and adsorption percentage of (**a**–**c**) S${}_{25}$ and (**d**–**f**) L${}_{25}$ proteins adsorbed by the three different polymer coatings (**a**,**d**) mushroom, (**b**,**e**) intermediate, (**c**,**f**) brush, versus time. These systems are free of A-beads, as indicated by the undoped chain in the graph, any attraction is caused by interactions with the bare implicit wall surface. Each panel explores the effect of the 8 different wall potentials ${}_{\epsilon }$W${}_{z_{c}}$ on the amount of adsorbed proteins.

Having calculated the size of each protein, we move on to study when we consider a protein to be adsorbed. For this study, a protein is considered adsorbed when it lies within the coating as a whole, or, in other words, when its center of mass is lower than the average height of the coating minus the radius of gyration of the protein, $R_{g,p}$. Based on these values, we plot the fraction of proteins adsorbed, $f_{p}^{\mathrm{ads}}$, versus time in Figure 6 and we see that there are two parameters affecting both the rate and the percentage of adsorption; the grafting density and the size of the proteins. A ’rainbow’ coloring scheme (shown in Table 2) is used in all of the following graphs for the effective interaction strength among proteins and the implicit wall at z=0.

For the two least attractive implicit wall surfaces (interactions ${}_{1}$W${}_{1}$ and ${}_{3}$W${}_{1}$) we do not find adsorption for any of the grafting densities, as expected. It is worthy to note here that for the mushroom, there is a considerably bigger amount of proteins going in and out of the coating as time goes by, depicted by a noisy adsorption curve. As soon as the effective interacting strength *I* increases (either by increasing the energy depth, $\epsilon_{\mathrm{wall}}$, or the cutoff distance, $r_{c,\mathrm{wall}}$), we notice a tendency for increase in the amount of adsorbed proteins, especially for the lower grafting densities - in most cases the higher the attraction the higher the adsorption percentage at a given time.

Qualitatively similar results were reported in the work of Yoshikawa et al. [101] for adsorption of Bovine Serum Albumin (BSA) proteins on poly(2-hydroxyethyl methacrylate) (PHEMA) brushes. Their adsorption curves are showcasing the effect of the grafting densities that we have in Figure 6. The average time for their experimental system to reach the final adsorption percentage is of the order of minutes. A BSA protein (which is slightly ellipsoidal [101]) is approximately of radius equal to $R_{g,\mathrm{BSA}}=2.5$ nm and has a molecular weight of $M_{w}=67$ kDa [101]. Using these values, the LJ unit length is of the order of 1 nm, and the LJ unit mass is roughly 1 kDa. Because our adsorption times are in the range of $10^{6}$ LJ units, the LJ unit time can be estimated as $10^{-4}$ s, which however gives rise to an unreasonably small LJ unit energy, small compared with $k_{B}T$. A direct one-to-one mapping of our model to protein adsorption is therefore not possible, while the molecular weight of the proteins and polymers used have a proportion that is again close to the experimental conditions [101].

The systems of the longer proteins (whose chain length is equal to 60 beads) make the aforementioned adsorption-related observations more apparent, as not only the adsorption percentage is lower compared to the systems of the shorter proteins at a given time, but also the rate of the adsorption decreases as can be clearly seen for the smaller grafting density, e.g., the mushroom. This can be justified by the bigger amount of hydrophobic beads causing a more often temporary ’grouping’ of proteins with one another, which was observed throughout a simulation. This temporary grouping of proteins might lead to a bigger excluded volume and at the same time a higher free energy barrier to be overcome in order to get adsorbed or just delay the adsorption process (as in [77]) till proteins ungroup once again.

#### 3.1.2. Effect of the Hydrophobicity Ratio

**Figure 7:**
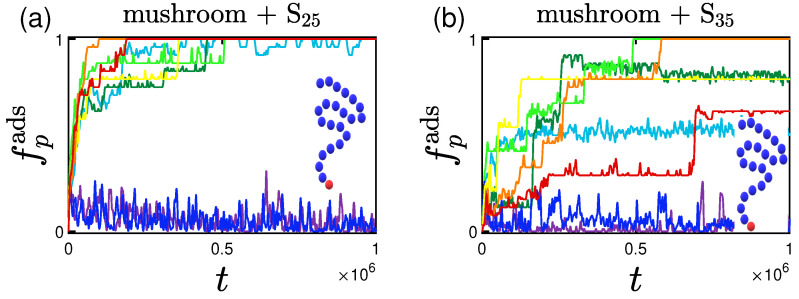
Effect of hydrophobicity for different attractive wall strengths (color code defined in Table 2). Rate and adsorption percentage of (**a**) S${}_{25}$ and (**b**) S${}_{35}$ proteins adsorbed by the mushrooms, in the absence of A-beads as indicated by the un-doped chain shown in the graph, versus time.

The hydrophobicity ratio is defined as the ratio of the hydrophobic beads towards the polar ones, as mentioned in Section 2.1.2. Two hydrophobicity ratios, hp, are studied: 25% and 35%. As seen in Table 4, the size of the proteins remained the same irrespective of the hydrophobicity ratio. The rate of adsorption on the other hand is certainly affected as can be seen in Figure 7; even though the adsorption curves have the same final values (fully adsorbed) with the ones of the lower hydrophobicity ratio, the pace of adsorption is much lower. This can be justified by the non-negligible interaction among proteins; more hydrophobic beads are ’exposed’ to the outer part of the proteins leading to stronger, and therefore more long-lasting, interactions. This leads to the creation of temporarily existing ’groups of proteins’, that in total have a bigger size than that of a single protein, leading to a lower rate of adsorption.

#### 3.1.3. Effect of the Position of Attractive A-Beads at the Backbone

In this subsection we study the case where we have an attractive bead A at the backbone of our polymer chain. This A-bead can be located either at the middle or at a random position of each polymeric chain tethered by one free end to the implicit wall at z=0.

**Figure 8:**
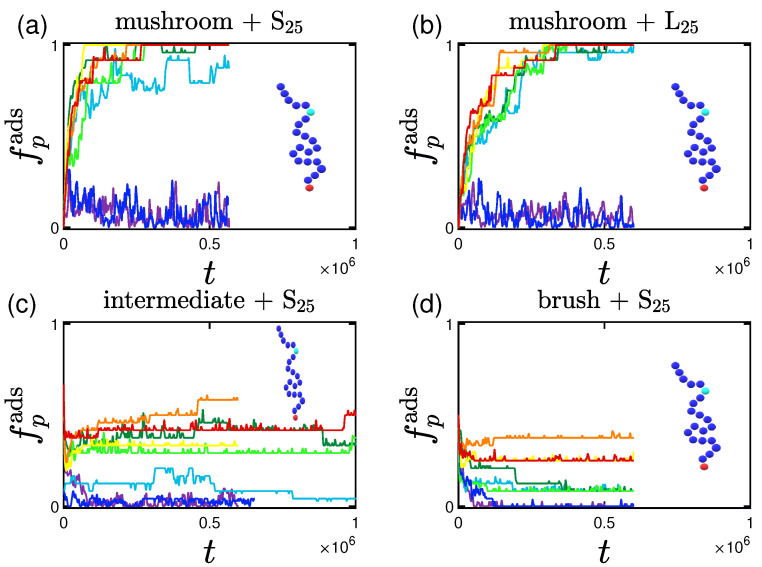
Effect of coating type and protein size for the randomly-doped polymer (chain doped by light blue A-beads of ${}_{1}$LJ${}_{1.5}$ at random backbone positions) for different wall attraction strengths (color code defined in Table 2). Adsorption percentage of (**a**) S${}_{25}$ and (**b**) L${}_{25}$ proteins adsorbed by the randomly doped mushrooms, as well as S${}_{25}$ adsorbed by the (**c**) intermediate and (**d**) brush coating, all versus time.

Concerning the amount or rate of adsorption we notice that there aren’t significant differences regarding the position of the attractive bead, just more intense fluctuations of the adsorption curve for the randomly placed A-bead. This can be due to the fact that there might be areas within the coating where there are no attractive beads at a specific height which leaves the coating with areas free from A-beads. Therefore whenever the excluded volume effect is strong enough, it leads to a tendency of the protein to reemerge at the top of the coating (as this would be more favorable energetically), since there are no neighboring attraction sites to keep the protein inside the coating. Some indicative plots of the percentage of adsorption for the randomly doped mushroom with A-beads of ${}_{1}$LJ${}_{1.5}$ interactions with the proteins can be found in Figure 8.

As far as it concerns the height, systems with A-beads on their backbone at the middle of the chain or at a random position with ${}_{1}$LJ${}_{1.5}$ interactions have similar height values to the undoped systems. This means that there is just a small increase of $h_{\max}$ and subsequently *R* as a side-effect of adsorption; more specifically for the mushroom there is an increase of $\Delta h_{\max}=+0.4$, for the intermediate an increase of $\Delta h_{\max}=+0.17$ and almost no increase for the brush as very few proteins are adsorbed. Therefore one can claim that there is greater extension for systems of higher protein adsorption, even though one would expect the extension to be higher for the higher grafting densities; which would be true if the same amount of proteins was adsorbed for all grafting densities.

For the more attractive ${}_{3}$LJ${}_{1.5}$ interactions though the opposite effect is observed. More specifically, the systems with I<1 for the random-bead system have lower $h_{\max}$ compared to the ones of higher *I* (about $\Delta h_{\max}=-0.4$ for the mushroom, $\Delta h_{\max}=-0.2$ for the intermediate and indifferent for the coating for S${}_{25}$; with the respective values for L${}_{25}$ being roughly $\Delta h_{\max}=-0.6$, $\Delta h_{\max}=-0.1$ and $\Delta h_{\max}=0$). This is caused because attractive A-beads tend to ’follow’ proteins that are heading towards the highly attractive implicit wall, an effect to be shown more explicitly in the following subsection for the system of attractive terminal A-beads.

#### 3.1.4. Effect of the Presence of Terminal Attractive A-Beads

**Figure 9:**
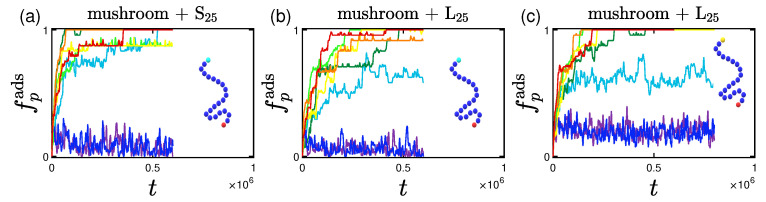
Effect of protein size and A-P interaction strength for terminal-doped mushrooms for different attractive wall strengths (color code defined in Table 2). Rate and adsorption percentage of (**a**) S${}_{25}$ and (**b**) L${}_{25}$ proteins adsorbed by the mushroom in the presence of regular terminal A-beads (chain doped by light blue A-beads of ${}_{1}$LJ${}_{1.5}$), versus time. In (**c**) we examine the adsorption also for more attractive terminal A-beads (chain doped by yellow A-beads of ${}_{3}$LJ${}_{1.5}$ ).

As seen in Figure 9, systems doped with attractive A-beads at the end of the chain showcase less steep adsorption curves the longer the protein and the lower the effective attraction strength of the wall, similar to the previously studied systems. For the system in Figure 9c, in which the terminal beads exert higher attraction forces on the hydrophilic beads of the proteins, we observe an overall increase at $f_{\mathrm{ads}}^{p}$ this time also for the cases of the lower *I* values. This should not surprise us, as the terminal beads of the mushroom have the space freedom to fit/move along with the proteins even under $h_{\max}$. The aforementioned system though is a bit different than the equivalent ones of the other cases as we will see when studying its density profile.

Important to be mentioned here is the fact that if we locate the terminal A-beads in space, we find that they lie at different heights partly depending on the attraction strength of the surface towards the protein, with the phenomenon being more obvious for the ${}_{3}$LJ${}_{1.5}$ effective interactions for the mushroom, which provides its individual chains a lot of free space to move. As a result, the high attraction among the terminal A-beads and proteins leads to the relocation of their average position closer to the implicit wall surface for I>1. For example, for L${}_{25}$ and ${}_{3}$LJ${}_{1.5}$, the average gets from 9.5 (which is similar to the equivalent $h_{\max}$) to a value around 6 for I>1 and where proteins lie close to the implicit wall. As a domino effect, there is a decrease in $h_{\max}$ from approximately 10.7 to 9.5 ($\Delta h_{\max}=-1.2$). The effect is even bigger if we think that the norm of adsorption is that the coating height is expected to increase. This might be contributing to a higher final adsorption than the one that would be expected for the equivalent system without the relocation of the attractive ’centers’ below $h_{\max}$.

#### 3.1.5. Effect of the Presence of the Ligands and Their Surface Density

**Figure 10:**
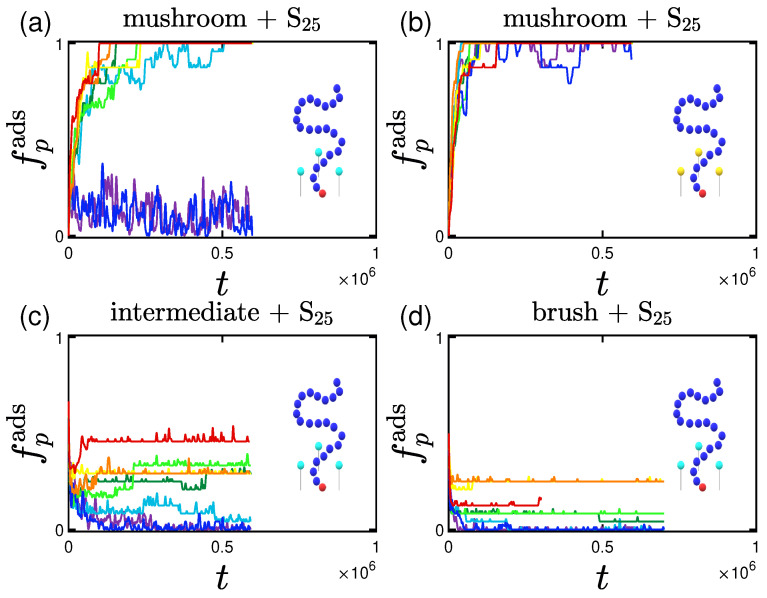
Effect of coating type for the system including high density ligands at z=3 at ligand grafting density $\sigma_{\mathrm{lig}}=0.023$ for different attractive wall strengths (same color code as in previous figures). Adsorption percentage of S${}_{25}$ proteins adsorbed by the mushrooms for (**a**) attractive ligand A-beads (light blue bead of ${}_{1}$LJ${}_{1.5}$) and (**b**) for more attractive ligand A-beads (yellow bead of ${}_{3}$LJ${}_{1.5}$), as well as S${}_{25}$ adsorbed by the (**c**) intermediate and (**d**) brush coating.

The last case we examine is the one of ligand A-beads immobile at a fixed height (z=3) for two ligand grafting densities ($\sigma_{\mathrm{lig}}=0.023$ and $\sigma_{\mathrm{lig}}=0.01$). Examining the rate and percentage of adsorption, we see in Figure 10 that in general the behavior is similar to the one of the system with the A-beads lying at the middle of the backbone, with $f_{\mathrm{ads}}^{p}$ being a bit lower for the intermediate and brush system, irrespective of the ligand grafting density.

Noteworthy is the main difference among the systems of ligand A-beads and backbone A-beads. For ${}_{3}$LJ${}_{1.5}$ all proteins get adsorbed even when there is no attraction by the implicit wall for the system with ligands. Obviously, for the systems with lower ligand density the phenomenon is definitely slower, and the curve is fluctuating more for the duration of the simulations studied, but it is obvious that the percentage will be way higher than the one of the equivalent system for the backbone A-bead case (Appendix A).

### 3.2. Protein Density Profiles

The protein density profiles are calculated based on the positions of the beads constituting proteins for the last 30% of our data. What is observed is that the more attractive the implicit wall the more proteins gather close to it, as expected. Similar profiles are retrieved for both short and long proteins as far as it concerns the wall attraction, with the concentration of the shorter proteins reaching slightly bigger values close to the wall surface.

#### 3.2.1. Effect of the Grafting Densities

**Figure 11:**
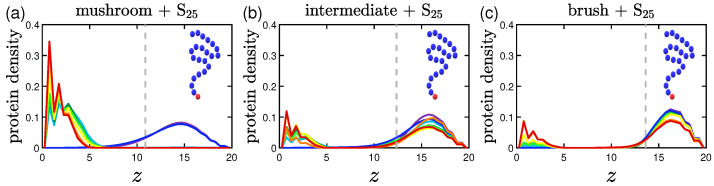
Effect of coating type on the protein density profile for S${}_{25}$ proteins adsorbed by the three different polymer coatings (**a**) mushroom, (**b**) intermediate and (**c**) brush (color code defined in Table 2). These systems are free of A-beads, which means that any attraction is caused by interactions with the bare implicit wall surface. Each panel explores the effect of the 8 different wall potentials ${}_{\epsilon }$W${}_{z_{c}}$ on the amount of adsorbed proteins. The dashed gray line is the average $h_{\max}$ of the coating for I>1. The wall at $z=L_{z}=20$ is purely repulsive for all bead types (${}_{1}$W${}_{..}$), that is why the density has a peak at large *z*.

Keeping the same color code to depict the several effective attraction strengths *I* of the implicit wall, we plot in Figure 11 the density profiles for the mushroom, the intermediate and the brush coating, for the small protein system (S${}_{25}$). The additional dashed gray line in the graphs stands for the respective averaged $h_{\max}$ for I>1. For wall attraction I<1 proteins tend to stay out of the coating (on the right of the dashed gray line), whilst away for the purely repulsive wall at z=20. For I>1, the highest peak of the curve is as close as possible to the attractive surface. This peak significantly decreases for the higher grafting densities due to excluded volume effects, for which we showed already that the amount of proteins adsorbed is low.

#### 3.2.2. Effect of the Hydrophobicity Ratio

**Figure 12:**
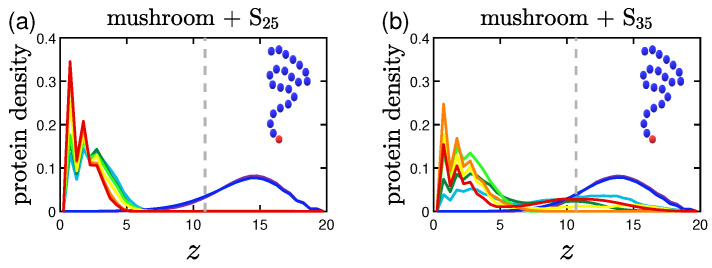
Effect of hydrophobicity, hp, on the protein density profile for (**a**) S${}_{25}$ and (**b**) S${}_{35}$ proteins adsorbed by the mushrooms, in the absence of A-beads as indicated by the sample chain of the graph (color code defined in Table 2). The dashed gray line is the average $h_{\max}$ of the coating for I>1.

The trend of the more hydrophobic systems to have lower $f_{\mathrm{ads}}^{p}$ at a given time is also apparent in the density profiles. Proteins density curves have not only lower peaks due to the lower $f_{\mathrm{ads}}^{p}$, but also wider ones close to the implicit wall surface at z=0. For the system of hp=35%, as seen in Figure 12, the curves take non-zero values throughout the coating, signifying that there are proteins to be found in all height within the coating. So, even when a protein is considered adsorbed according to our definition, it can lie at various heights of the coating.

#### 3.2.3. Effect of the Position of the Attractive A-Beads at the Backbone

**Figure 13:**
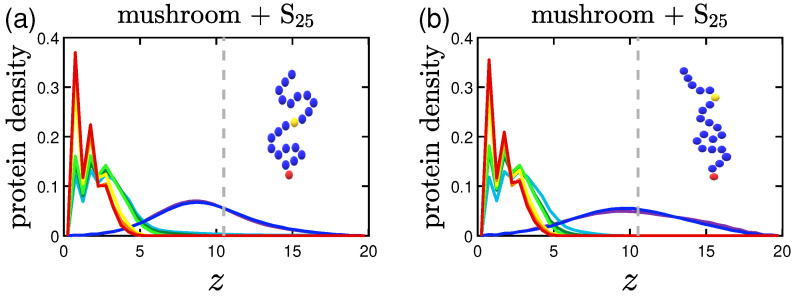
Effect of the position of the attractive backbone beads embedded in mushrooms on the protein density profile for S${}_{25}$ proteins adsorbed by highly attractive (**a**) middle-doped and (**b**) randomly-doped mushrooms (yellow beads of ${}_{3}$LJ${}_{1.5}$). The dashed gray line is the average $h_{\max}$ for I>1.

A Backbone beads lying in the middle of coating chain show similar results to A backbone beads at random positions within the chain concerning protein density profiles, with their main difference being where the peak of the curve lies for I<1; the systems doped with A-beads at the middle of the chain (especially the ones with ${}_{3}$LJ${}_{1.5}$) have their peak for I<1 close to the position of the A-beads, while the random ones seem to have a wider distribution with a lower peak (see Figure 13. The opposite phenomenon is observed for the higher grafting densities of the intermediate and the brush coating; here the distributions of the middle A-bead systems are wider compared to the random ones, show higher peaks outside of the coating and lower ones close to the attractive implicit wall. The attractive sites that lie at various coating heights are easing out the adsorption of proteins at certain parts of the coating (this is why there were also more fluctuations of the adsorption curve) and subsequently the overall process, which as seen can be very useful if one wants to achieve protein adsorption on brushes without our attractive sites being ’screened’ by the density of the brush.

#### 3.2.4. Effect of the Presence of Terminal Attractive A-Beads

**Figure 14:**
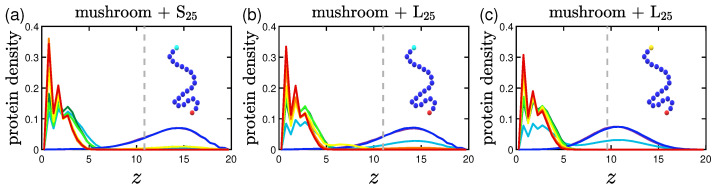
Effect of protein size and A-P interaction strength on the protein density profile for terminal-doped mushrooms. Protein bead number density profiles of (**a**) S${}_{25}$ and (**b**) L${}_{25}$ proteins adsorbed by the mushroom in the presence of regular terminal A-beads (light blue beads on the single chain with ${}_{1}$LJ${}_{1.5}$). In (**c**) we examine the adsorption also for more attractive terminal A-beads (yellow beads on the backbone of ${}_{3}$LJ${}_{1.5}$). The dashed gray line is the average $h_{\max}$ for I>1.

For a terminally-doped mushroom, the main tendencies remain with the short S${}_{25}$ proteins being close to the wall for I<1, while the longer ones L${}_{25}$ showing a slightly wider distribution and for I≈1 some of them prefer to stay at the top of the coating, as seen in Figure 14a,b. In Figure 14b,c there is a noticeable relocation of the maximum of the peak closer to the terminal A-beads, with the relocation being bigger the higher the strength of the attractive interactions ${}_{1,3}$LJ${}_{1.5}$.

What needs to be noted here is the behavior already described in Section 3.1.4. Keeping in mind that in Figure 14 the gray dashed line corresponds to the averaged $h_{\max}$ for I>1, we have to mention the effect for Figure 14c: the maximum of the peak is in reality at the height of the polymer brush for I<1 due to the highly attractive interactions ${}_{3}$LJ${}_{1.5}$ exerted by the terminal A-beads towards the proteins ($h_{\max}$ is 10.7 for I<1 and 9.5 for I>1).

#### 3.2.5. Effect of the Presence of the Ligands and Their Surface Density

**Figure 15:**
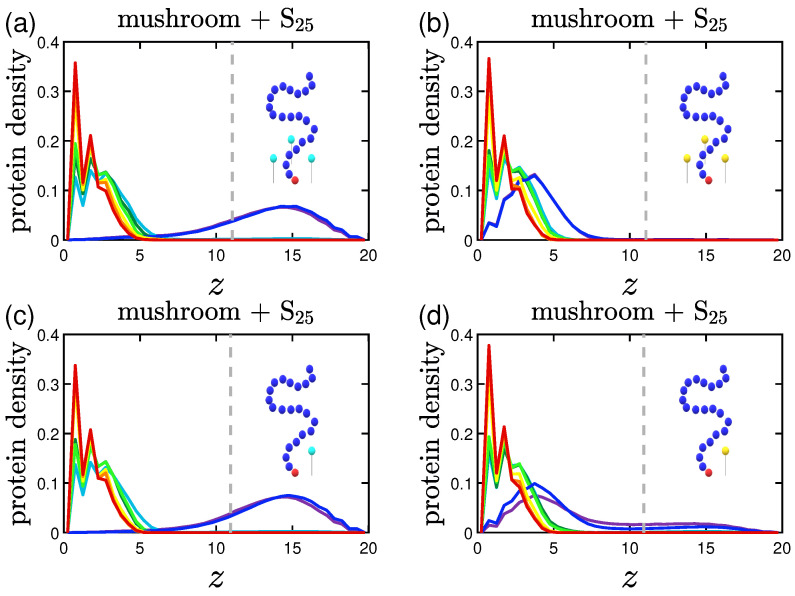
Effect of ligand surface density (vertical) and ligand strength (horizontal) on the protein density profile for the system including S${}_{25}$ proteins adsorbed by the mushrooms, for different attractive wall strengths. For the higher ligand surface density $\sigma_{\mathrm{lig}}=0.023$ (indicated by three ligand beads by the chain) we depict the protein density profile of (**a**) regular attractive ligand A-beads (light blue bead of ${}_{1}$LJ${}_{1.5}$) and (**b**) strongly attractive ligand A-beads (yellow bead of ${}_{3}$LJ${}_{1.5}$), and the respective protein density profiles for the lower ligand surface density $\sigma_{\mathrm{lig}}=0.01$ (indicated by one ligand bead by the chain) in (**c**,**d**). The dashed gray line is the average brush height for I>1.

The protein density profiles of mushrooms with attractive A ligand beads immobile at z=3 have many similarities to the systems doped with attractive A backbone beads. What differs is the behavior of the systems of highly attractive ligands of ${}_{3}$LJ${}_{1.5}$, as one can see in Figure 15. The density profiles of these systems indicate that even when the wall attraction is very weak (I<1), there is a high adsorption of proteins as already observed in Section 3.1.5. The phenomenon is observed no matter what the ligand density is; with the result being more intense for the higher ligand density system. The peaks are lower also for the L${}_{25}$ proteins due to the higher amount of hydrophobic beads. Although for the denser coatings, there is some sort of screening of the attractive A ligand beads because of the density of the coating, and thus, along with the denser coating itself, there is only partial adsorption of proteins.

### 3.3. Shape of the Proteins

**Figure 16:**
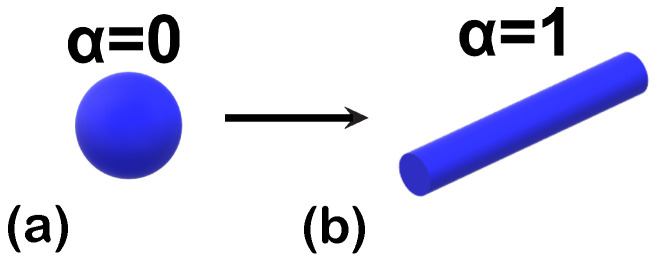
Optical illustration of the potential asphericity values, where α=0 represents a spherical object (**a**) and α=1 represents a rodlike object (**b**).

Next we examine the shape of the proteins by comparing their asphericity values. The asphericity of a single protein, α, is defined as [102,103,104,105].

(7) $$\alpha =\frac{{\left(R_{1}-R_{2}\right)}^{2}+{\left(R_{2}-R_{3}\right)}^{2}+{\left(R_{3}-R_{1}\right)}^{2}}{2R_{g,p}^{4}}$$

where $R_{1}$, $R_{2}$ and $R_{3}$ are the three diagonal elements of the gyration tensor, $R_{g,p}$, of a protein. According to this definition, α=0 applies to spherically symmetric objects and α=1 is for perfectly elongated (rodlike) shaped objects (Figure 16). A potential interpretation of the asphericity values is that the closer proteins are to a rodlike shape, the most likely is the protein unfolded. As we will see later on in this section, our proteins tend to keep their spherical shape (i.e., they are in a folded globular state) as their small asphericity values indicate, just slightly changing depending on their surroundings. This is inline with the observations and experiments of Anfinsen et al. [98,106], who found that a globular protein tends to keep its shape with small fluctuations about its most stable conformation [107].

**Figure 17:**
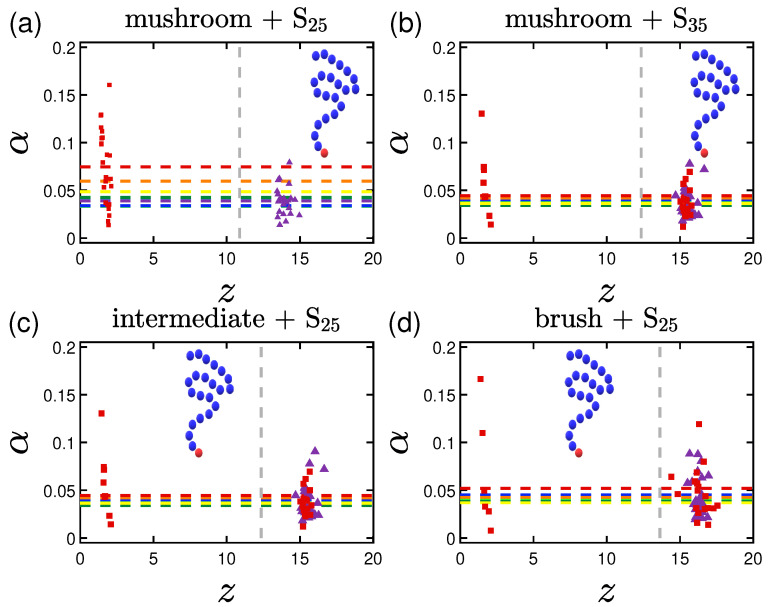
Effect of the coating and the hydrophobicity on protein shape. Asphericity of (**a**) S${}_{25}$ and (**b**) S${}_{35}$ proteins adsorbed by undoped mushrooms with no special A-beads. In (**c**,**d**) the asphericity of S${}_{25}$ is depicted for the undoped intermediate and undoped brush coating, respectively. α stands for the time-averaged asphericity of a single protein, for the last 30% of our data. The horizontal dashed lines represent the average asphericity values following the coloring scheme that was adopted in the previous sections for the wall attraction. Purple triangles are the average positions for each of the 24 proteins for ${}_{1}$W${}_{1}$ and with red squares for ${}_{3}$W${}_{2.5}$ (Table 2).

Therefore the initial asphericity values (as seen in Figure 17 for the S proteins) are as expected close to 0 as we start from pre-equilibrated protein conformations, with a tendency to have a slightly non-spherical shape for the highly adsorbed systems of the mushrooms. Similar findings apply also to the big L protein systems.

A general observation regarding the shape of the protein is that the denser and less attractive the system, the more spherical the protein. In other words, for the higher grafting densities of the coating, minor change on the average value of asphericity is seen (Figure 17), no matter what other parameters are changed (as for example the existence of highly attractive A-beads). Following the same coloring scheme for the wall interactions, we plot with dashed horizontal lines the average values for the 24 proteins over the last 30% of the data of our simulations. On top of that, we depict the average positions of the $G_{p}$ proteins in the *z*-dimension plotted against their asphericity values with purple triangles for ${}_{1}$W${}_{1}$ and with red squares for ${}_{3}$W${}_{2.5}$, see Table 2. Once again the vertical dashed gray line represents the average $h_{\max}$ for I>1. Comparing these aspherities, we find that the more hydrophobic the system, the more spherical the protein, regardless of the strength of the wall attraction (Figure 17).

Similar results we observe for the systems with longer proteins. Here, trends are slightly more apparent, probably due to the number of beads being attracted by the surface. The difference in the values is also increasing, when we are studying systems of highly attractive backbone A-beads (no matter where they are located in the chain) or systems with ligand A-beads.

**Figure 18:**
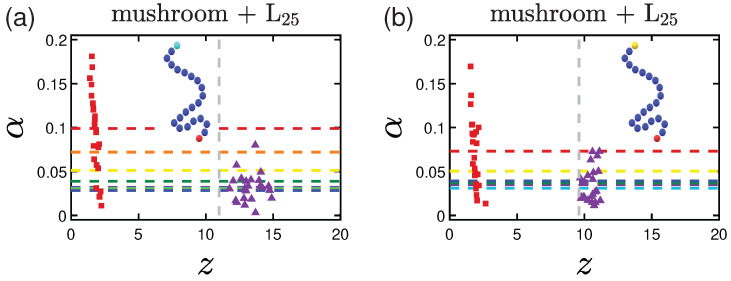
Effect of the terminal-doped mushrooms. Asphericity of L${}_{25}$ proteins adsorbed by mushrooms for (**a**) attractive terminal A-beads (${}_{1}$LJ${}_{1.5}$) and (**b**) more attractive terminal A-beads (${}_{3}$LJ${}_{1.5}$). The horizontal dashed lines represent the average asphericity values following the coloring scheme that was adopted in the previous sections for the wall attraction. Purple triangles are the average positions for each of the 24 proteins for ${}_{1}$W${}_{1}$ and with red squares for ${}_{3}$W${}_{2.5}$.

An interesting phenomenon is observed in Figure 18 for the case of the terminally-doped coatings. In this case, the previously mentioned effect of higher asphericity values for the L proteins is vanishing for the ${}_{3}$LJ${}_{1.5}$ interaction strength, as the proteins are, most likely, slightly pulled away from the surface due to the attractive terminal R-beads being present at a height around z=6, instead of being at a height close to the value of the end-to-end distance, *R*. This is happening due to the fact that the terminal beads have ’followed’ the proteins towards the inner part of the coating due to the highly attractive forces among these terminal A-beads and the hydrophobic part of the protein.

### 3.4. Potential of Mean Force (PMF)

**Figure 19:**
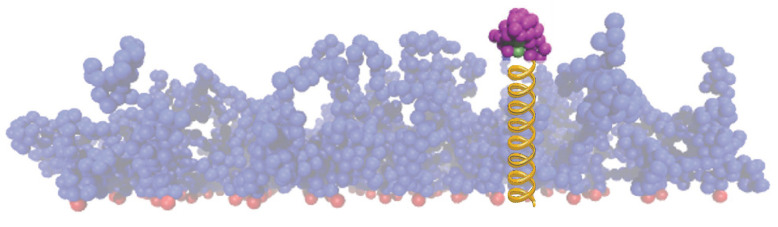
Brush configuration for a mushroom coating without special A-beads for a single protein tethered to the wall by a harmonic spring of yellow color (the coating is more transparent in order to help the eye of the reader).

To retrieve the free energy of adsorption for our systems, we calculated the one-dimensional potential of mean force (PMF) of a single protein combined to umbrella sampling, using $N_{w}$ umbrella windows, and the weighted histogram analysis method (WHAM) along the reaction coordinate *z*. To this end we used Steered Molecular Dynamics (SMD) simulations, in which the center of mass of the protein, initially located outside the brush, is tethered to the implicit wall surface at z=0 via a harmonic spring characterized by its variable stiffness $k_{\mathrm{spring},j}$ for $j=1,2,\cdots ,N_{w}$, and equilibrium length $l_{\mathrm{spring}}$ (Figure 19). No constraints were applied along the lateral dimensions [108].

The biasing spring potential $w_{j}\left(z\right)$ for the *j*th umbrella window is

(8) $$w_{j}\left(z\right)=\frac{k_{\mathrm{spring},j}}{2}{\left(z-l_{\mathrm{spring}}\right)}^{2},$$

where *z* is the reaction coordinate for the PMF calculation.

For the umbrella sampling simulations a proper range of $k_{\mathrm{spring},j}$ must be chosen, while the spring equilibrium length we set to $l_{\mathrm{spring}}=1$, i.e., roughly equal to the gyration radius of the protein. We find that beyond $k_{\mathrm{spring},j}=0.3$, the stationary spring extension in *z*-direction tends to remain unchanged as the protein has reached the tethering surface. Therefore for our umbrella sampling $k_{\mathrm{spring}}$ is equidistantly varied from 0.0 to 0.3, i.e., $k_{\mathrm{spring},j}=j\Delta k_{\mathrm{spring}}$ with $\Delta k_{\mathrm{spring}}=0.005$, leading to $N_{w}=61$ umbrella windows. Each umbrella window runs for a duration of t=500 (saving every 10 time steps leading to $N_{i}=10^{4}$ snapshots for each window), using the exact same equations and conditions for the previously described systems, apart from the added spring force.

The recorded trajectories of the spring extensions within each window are post-processed via the WHAM equations proposed by Grossfield [109,110]. WHAM takes into account the statistical uncertainty of the unbiased probability distribution P(z) of *z*-coordinates in order to compute the PMF(z) that corresponds to the smallest uncertainty. This is done through the iterative solution of the following implicit equation

(9) $$P\left(z\right)=\frac{\sum_{j=1}^{N_{w}}g_{j}^{-1}h_{j}\left(z\right)}{\sum_{j=1}^{N_{w}}N_{j}g_{j}^{-1}\exp\left[-\beta \left(w_{j}\left(z\right)-f_{j}\right)\right]}$$

for P(z), where $f_{j}$ is expressed in terms of P(z) via

(10) $$\exp\left(-\beta f_{j}\right)=\int_{0}^{L_{z}}\exp\left(-\beta w_{j}\left(z\right)\right)P\left(z\right)dz,$$

and where $h_{j}\left(z\right)$ is the histogram of $N_{j}$*z*-coordinates from window *j*, $g_{j}={\left(1+2\tau_{j}\right)}^{-1}$ is an overall factor determined by the integrated autocorrelation time $\tau_{j}$ for window *j*, and $f_{j}$ is the free energy of the system described by the Hamiltonian, within window *j*. The limit $L_{z}$ of the integral is due to the non-periodic dimensions of the simulation box along the reaction coordinate. The PMF is the given by

(11) $$\mathrm{PMF}\left(z\right)=\frac{1}{\beta }\ln\frac{P(z)}{P(z_{0})},$$

where $P(z_{0})$ is an arbitrarily chosen reference point ensuring PMF$(z_{0})=0$.

**Figure 20:**
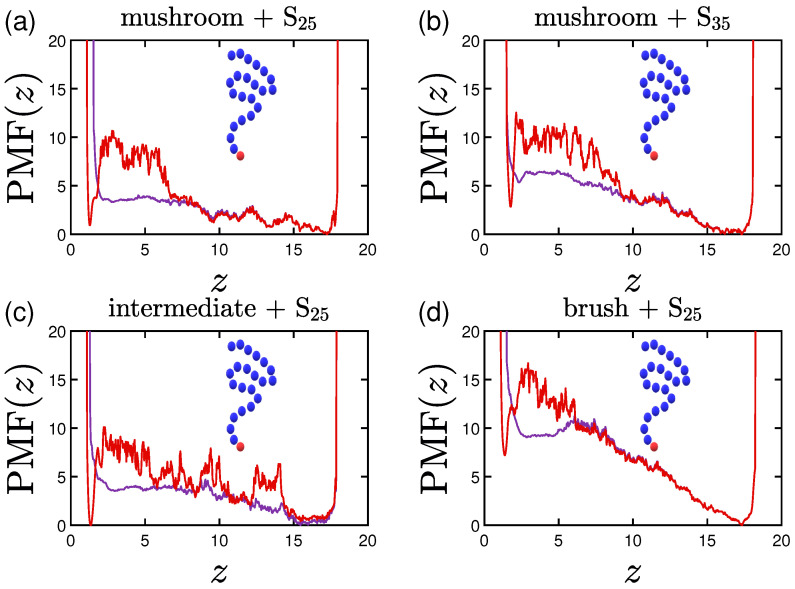
Effect of the coating and the hydrophobicity on PMF(z). The PMF of a single (**a**) S${}_{25}$ and (**b**) S${}_{35}$ protein adsorbed by undoped mushrooms with no special A-beads. In (**c**,**d**) the PMF of a single S${}_{25}$ protein is depicted for the undoped intermediate and undoped brush coating, respectively. α stands for the time-averaged asphericity of a single protein, for the last 30% of our data. As for the previous plots, purple lines depict PMF(z) curves for ${}_{1}$W${}_{1}$ and red ones for ${}_{3}$W${}_{2.5}$ (Table 2).

**Figure 21:**
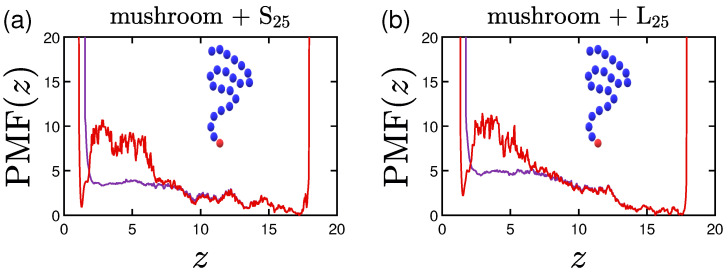
Effect of the molecular weight of the protein on its PMF, for an undoped mushroom. (**a**) S${}_{25}$ and (**b**) L${}_{25}$ protein.

**Figure 22:**
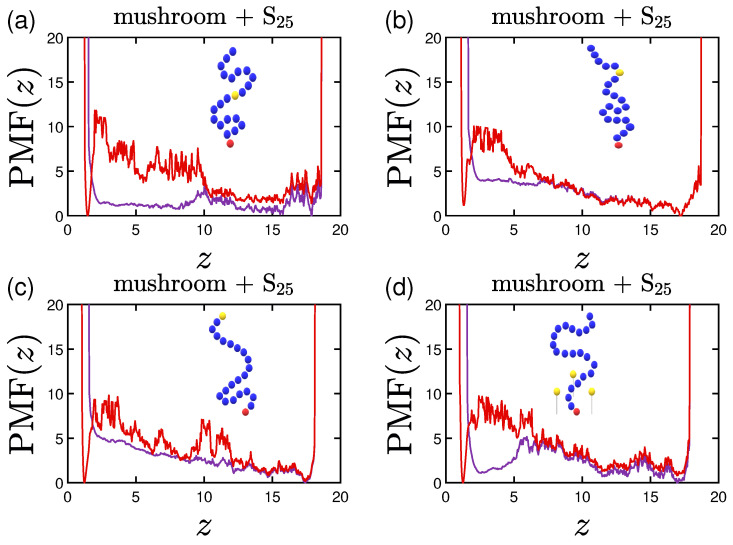
Effect of polymer doping on the PMF(z) of a S${}_{25}$ protein adsorbed by mushrooms for (**a**) attractive middle, (**b**) attractive random, (**c**) attractive terminal and (**d**) attractive ligand A-beads (all of ${}_{3}$LJ${}_{1.5}$ attraction). The purple lines represent the lowest wall attraction and the red ones the highest wall attraction, following the previous coloring scheme.

Following the aforementioned coloring scheme, we plot in Figure 20, Figure 21 and Figure 22 with purple the single protein PMF for the system with ${}_{1}$W${}_{1}$ wall attraction and with red the one employing ${}_{3}$W${}_{2.5}$. As soon as the protein reaches the coating, the force needed to reach the implicit wall surface is growing due to the ’resistance’ caused by the chains of the coating. The higher the grafting density of the coating, the less prone is the protein to stay inside the coating, especially for the less attractive implicit wall cases. The curves are steep close to the two wall surfaces, as the protein can never reach them. There is also a minimum close to the lower wall for the ${}_{3}$W${}_{2.5}$ cases due to the wall attraction, which is slightly varied for the different protein configurations. Validating our aforementioned findings, the higher the hydrophobicity ratio or the molecular weight the protein, the higher is the energy barrier needed to be overcome in order for the protein to reach the wall at z=0 (Figure 20 and Figure 21). The high peaks in the vicinity of the wall appear as soon as the protein is within the range of the cutoff distance for the attraction of the wall.

Noteworthy is the PMF(z) behavior for the doped coatings with attractive beads of ${}_{3}$LJ${}_{1.5}$. For the case of a middle A-doped coating (Figure 22a), even for the lower wall attraction, the existence of the special A-beads facilitates low energies for the protein adsorption in the vicinity of these beads, which validates our findings from the previous sections. Since the beads are mobile in this case, the range seems to be wider compared to the case of the ligand in Figure 22d. The randomly placed A-beads exhibit a similar effect, but less profound, while for the terminal A-beads one can clearly see that there is a strong preference for the protein to stay close to them and out of the brush, especially for the least attractive wall.

## 4. Conclusions

The proposed polymer+protein model is qualitatively predicting the primary, secondary, and ternary adsorption tendencies of interacting globular proteins under certain conditions, which we explored. Using the results obtained here during variation of several relevant system parameters, one may combine the various cases to tune a system of interest after calibration, so that the final amount of adsorption will be close to the desired one, depending on the system’s requirements and the technical/chemical characteristics as well as the conditions of the surrounding environment. For example, by tuning the grafting density, the model predicts whether proteins are preferably adsorbed or not, and could insofar support experimental studies regarding the fouling/anti-fouling possibilities of a polymer coating. When designing the wanted characteristics one has to take into consideration that protein adsorption is causing crowding to the polymer coating leading to a further elongation (swelling) of the polymer chains when increasing the grafting density of the coating, as validated by our study. This swelling is energetically favorable upon overcoming a certain energy barrier; PMF curves showcase this effect for systems in which there is (i) an attraction of proteins towards the implicit wall surface (primary adsorption) or (ii) some special sites that attract the hydrophilic part of the protein (such as attractive backbone or terminal beads or ligands). The more attractive these sites are, the more intense the phenomenon and the higher the rate of secondary adsorption. For the cases of the attractive towards the proteins implicit wall, the proteins tend to accumulate close to the tethering surface. This is quantified by peak heights of their density profiles close to the surface; the peak becomes generally narrower and more pronounced when the attraction increases. The existence of these special beads along our CG polymer chains is signifying an increase to the rate and percentage of adsorption, while the presence of highly attractive backbone beads is causing some ’pulling’ of the proteins away from the attractive surface, when at the same time the height of the brush tends to slightly decrease. The attractive beads tend to ’follow’ the protein to areas closer to the wall (for the most attractive protein-wall interactions). The most extreme case where such a phenomenon is observed here is for the attractive terminal beads. We find that ligands exhibit more or less the same efficiency as the attractive backbone beads, but with higher intensity, as adsorption is now observed for the least attractive surfaces as well.

As for the hydrophobicity effect to adsorption and the effect of the size of the protein, we can state that adsorption is seen to become significantly slower as proteins tend to interact more with one another. They create small temporary protein agglomerates, which are not as easy to adsorb. In most cases studied, our proteins tend to stay globular, deviating only slightly from their near-spherical shape for the highly attractive implicit wall, with the phenomenon being more intense for the bigger proteins.

To our knowledge, there are no CG molecular dynamics studies available for comparison that take into consideration protein-protein interactions, while fully atomistic studies remain unfeasible at present for several reasons [111]. There is a lattice Monte Carlo (MC) study that includes interactions among proteins. It reproduces static results similar to ours for a specific system [57]. Another MC study of the adsorption of single peptides and their aggregates [77] further validates our model causing the delay of the adsorption due to aggregation. Other than these investigations, most of the current studies either ignore the protein-protein interaction [112] or prefer to study a single protein (atomistic or CG) over a polymeric coating or a surface in general [113,114].

What remains to be explored is broader comparison with experimental results upon varying the polymerization degree of our polymer and/or protein chains to match the experiment and potentially bigger proteins to examine the margin of applicability of the model. An additional further step could be the investigation of the characteristic folding/unfolding of the proteins during adsorption, either by loosening the current hydrophobic interactions or by incorporating additional interactions, such as explicit electrostatic interactions of variable strength.

## Figures and Tables

**Table 1:** This table summarizes the interactions between the various bead types and the unstructured wall surface: R (repulsive polymer bead), w (water bead), H (hydrophobic protein bead), P (polar protein bead), and A (attractive polymer or ligand bead). Polymer R beads are depicted with red when they are tethered to the implicit wall surface and with blue when they are not. An A-bead (either mobile as part of the backbone or immobile at a fixed height as a ligand bead) is of light blue color for ${}_{1}$LJ${}_{1.5}$ and of yellow color for ${}_{3}$LJ${}_{1.5}$ interactions. Proteins and flexible polymer chains have identical bond length potentials. Each polymer brush chain has N=50 beads, while proteins have $N_{p}\in \left\{40,60\right\}$ beads and two different hydrophobicity ratios hp ∈{25%,35%}. Three different tethering densities σ, 6 different placements of A-beads (none, middle, random, end, ligand at low density, ligand at high density), 8 different attractive wall types ${}_{\epsilon }$W${}_{z_{c}}$ are studied, while the bead number density n=0.65 and system volume remain fixed. Interactions with the wall are described by the 9-3 potential ${}_{\epsilon }$W${}_{z_{c}}$ with ϵ∈{1,3} and $z_{c}\in \left\{1.0,1.5,2.0,2.5\right\}$,the purely repulsive wall is denoted by RW. Beads interact via the ${}_{\epsilon }$LJ${}_{r_{c}}$ potential; the purely repulsive WCA potential equals a LJ potential with ϵ=1 and $r_{c}=2^{1/6}$. The bead coloring scheme is mentioned in the last column.

	R-Bead	w-Bead	H-Bead	P-Bead	A-Bead	Wall	Color
R-bead	WCA	WCA	WCA	WCA	WCA	RW	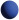 , 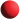
w-bead	WCA	WCA	WCA	WCA	WCA	RW	—
H-bead	WCA	WCA	${}_{120}$LJ${}_{1.19}$	WCA	WCA	${}_{\left\{1,3\right\}}$W${}_{\left\{1.0:0.5:2.5\right\}}$	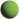
P-bead	WCA	WCA	WCA	WCA	${}_{\left\{1,3\right\}}$LJ${}_{1.5}$	${}_{\left\{1,3\right\}}$LJ${}_{\left\{1.0:0.5:2.5\right\}}$	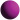
A-bead	WCA	WCA	WCA	${}_{\left\{1,3\right\}}$LJ${}_{1.5}$	WCA	RW	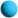 , 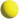

**Table 2:** Color code used throughout for the 8 wall types characterized by their interaction ${}_{\epsilon }$W${}_{z_{c}}$ with all beads. Here, ϵ is the depth of the potential and $z_{c}$ the cutoff distance. The list is sorted by the effective interaction strength, $I\equiv \int_{0}^{z_{c}}\exp\left[-W\left(z\right)/k_{B}T\right]dz$.

Wall	${}_{1}$W${}_{1}$	${}_{3}$W${}_{1}$	${}_{1}$W${}_{1.5}$	${}_{1}$W${}_{2}$	${}_{1}$W${}_{2.5}$	${}_{3}$W${}_{1.5}$	${}_{3}$W${}_{2}$	${}_{3}$W${}_{2.5}$
color	purple	blue	light blue	green	light green	yellow	orange	red
	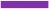	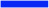	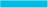	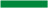	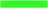	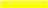	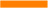	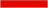
*I*	0.307	0.349	1.184	1.937	2.572	3.088	5.734	7.422

**Table 3:** Linear polymers tethered on a planar surface, surrounded by water, in the absence of proteins. Stationary ensemble values for the coating height $h_{\max}$, gyration radius $R_{g}$ and end-to-end distance *R* (as defined in the text) of the polymer coating for the last 30% of the data of each simulation (up to at least $t=5\times 10^{5}$), saving data-files each 500 time units. The dry coating height is $h_{\mathrm{dry}}=\sigma N/n$. For the corresponding bulk polymer, $\langle R^{2}\rangle \approx 1.64\left(N-1\right)=9.05$ and $\langle R_{g}^{2}\rangle \approx 3.70$ [81]. All reported numbers are in LJ units.

Polymer Coating	σ	*N*	*G*	$h_{\mathrm{dry}}$	$h_{\max}$	$R_{g}$	*R*	$\sigma \langle R_{g}^{2}\rangle$
mushroom	0.023	50	81	1.78	10.8	4.5	9.0	0.5
intermediate	0.056	50	196	4.31	12.1	4.7	10.5	1.3
brush	0.087	50	306	6.73	13.4	5.0	11.9	2.2

**Table 4:** Conformational properties of the four types of short (S) and long (L) proteins dissolved in water at a protein concentration $c_{p}$ (mass per volume) typical for our setup ($c_{p}\in \left[0.0007,0.001\right]\times N_{p}$). We find that the results do not depend on concentration over the mentioned range. The table collects the average protein gyration radius $R_{g,p}$ and end-to-end distance $R_{p}$ of the $G_{p}$ proteins for each type, using the last 30% of the data of each simulation (up to at least $t=5\times 10^{5}$), saving data-files each 500 time units. For the corresponding bulk polymer, where all H- and P-beads are replaced by repulsive R beads, $\langle R^{2}\rangle \approx 8.10$ (9.92) and $\langle R_{g}^{2}\rangle \approx 3.31$ (4.05) for $N_{p}=40$ (60) [81].

Protein	hp	$N_{p}$	$G_{p}$	$R_{g,p}$	$R_{p}$
S${}_{25}$-protein	25%	40	24	1.9	2.9
L${}_{25}$-protein	25%	60	24	2.1	2.7
S${}_{35}$-protein	35%	40	24	1.9	3.1
L${}_{35}$-protein	35%	60	24	2.1	2.5

**Figure A1:**
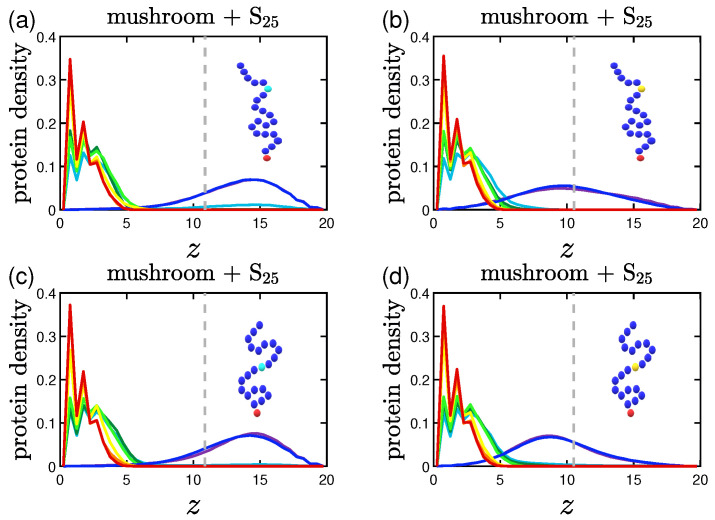
Effect of the position of the backbone A-beads embedded in mushrooms on the protein density profile for S${}_{25}$ proteins adsorbed by (**a**) attractive randomly-doped (light blue beads of ${}_{1}$LJ${}_{1.5}$), (**b**) highly attractive randomly-doped mushrooms (yellow beads of ${}_{3}$LJ${}_{1.5}$) and the middle-doped equivalents in (**c**,**d**). The dashed gray line is the average $h_{\max}$ for I>1.

**Figure A2:**
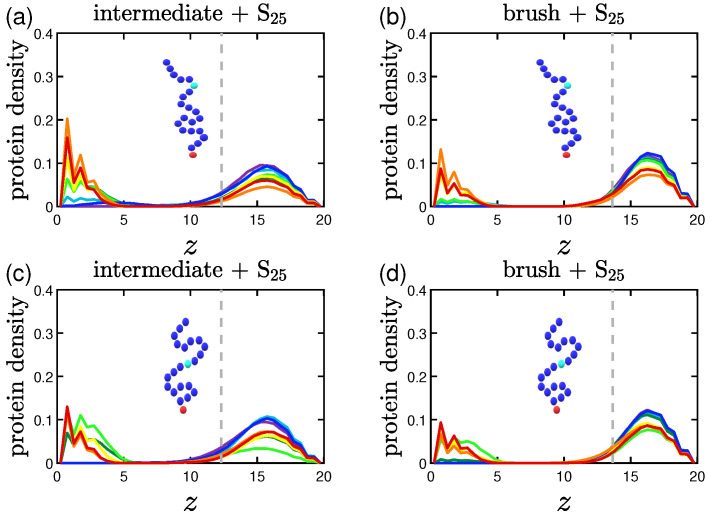
Effect of the grafting density for the backbone A-beads embedded in mushrooms on the protein density profile for S${}_{25}$ proteins adsorbed by attractive randomly-doped (light blue beads of ${}_{1}$LJ${}_{1.5}$) for (**a**) intermediate systems, (**b**) brush systems and in (**c**,**d**) their equivalents for the middle-doped cases. The dashed gray line is the average $h_{\max}$ for I>1.

**Figure A3:**
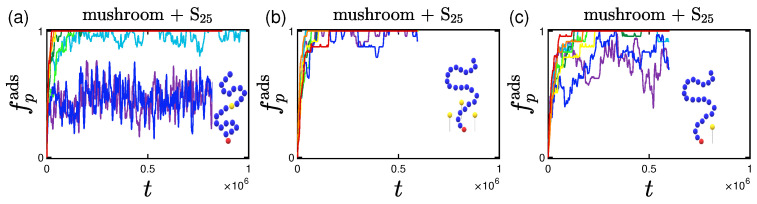
Effect of mobility of A-beads for a mushroom through comparison of mobile A-beads placed at the middle of the backbone and immobile A ligands fixed at z=3 for different attractive wall strengths (same color code as in previous figures). Adsorption percentage of S${}_{25}$ proteins adsorbed by the mushrooms for (**a**) mobile attractive middle A-beads (yellow bead of ${}_{3}$LJ${}_{1.5}$), (**b**) for immobile ligand A-beads for ligand grafting density $\sigma_{\mathrm{lig}}=0.023$ (yellow bead of ${}_{3}$LJ${}_{1.5}$) and (**c**) immobile ligand A-beads for ligand grafting density $\sigma_{\mathrm{lig}}=0.01$.

**Figure A4:**
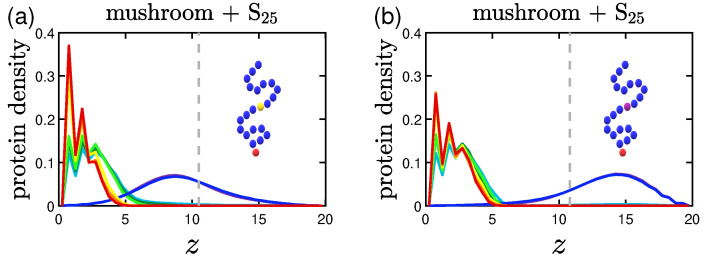
Effect of hydrophobicity of A-beads for a mushroom through comparison of hydrophilic and hydrophobic A-beads placed at the middle of the backbone for different attractive wall strengths (same color code as in previous figures). Protein density of S${}_{25}$ proteins adsorbed by the mushrooms for (**a**) hydrophilic attractive middle A-beads (yellow bead of ${}_{3}$LJ${}_{1.5}$) and (**b**) for hydrophobic attractive middle A-beads (purple bead of ${}_{3}$LJ${}_{1.5}$).

**Figure A5:**
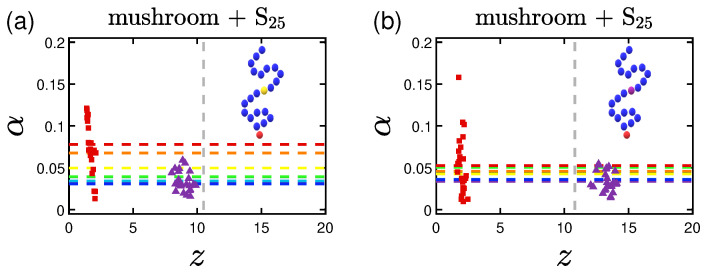
Effect of hydrophobicity of A-beads for a mushroom through comparison of hydrophilic and hydrophobic A-beads placed at the middle of the backbone for different attractive wall strengths (same color code as in previous figures). Asphericity of S${}_{25}$ proteins adsorbed by the mushrooms for (**a**) hydrophilic attractive middle A-beads (yellow bead of ${}_{3}$LJ${}_{1.5}$) and (**b**) for hydrophobic attractive middle A-beads (purple bead of ${}_{3}$LJ${}_{1.5}$).

## Data Availability

The data shown in this manuscript will become available at ETH Research collection (https://doi.org/10.3929/ethz-b-000612837, accessible from 25 May 2023 onwards).

## Appendix A. Supplementary Data for the Effect of A-Bead Position at the Backbone

To showcase the effect we mentioned in Section 3.2.3, we provide supplementary Figure A1. The peaks of the curves for I<1 are indeed close to the position of the A-beads, while the random ones seem to have a wider distribution with a lower peak, especially for the highly attractive A-bead systems with ${}_{3}$LJ${}_{1.5}$ that we have shown before.

In addition, we provide here the figures of the intermediate and the brush coating (Figure A2). The distributions of the middle A-bead systems are wider compared to the random ones, and show higher peaks outside of the coating and lower ones close to the attractive implicit wall.

## Appendix B. Mobile versus Immobile A-Beads

For a better understanding of the adsorption behavior of the several systems Figure A3 compares the effect of mobile A-bead of ${}_{3}$LJ${}_{1.5}$ placed in the middle of the backbone chain to an immobile A ligand bead of the same interaction strength fixed at z=3 (ligand grafting densities $\sigma_{\mathrm{lig}}=0.023$ and $\sigma_{\mathrm{lig}}=0.01$, various attractive wall strengths). Based on these data, the systems containing immobile A-beads exhibit much higher final adsorption, especially for the systems of low effective attractive strength of the wall, *I*, irrespective of the ligand density.

## Appendix C. Hydrophobic versus Hydrophilic A-Beads

To understand what the difference of having a system of hydrophobic instead of hydrophilic A-beads would be, we studied the effect for a system doped with middle A-beads at the backbone chain. Therefore, we depict the protein density for hydrophilic A middle beads and hydrophobic ones in Figure A4. Systems with hydrophobic A-beads tend to adsorb proteins less, which is expressed through wider density profiles of lower peaks. For the highly attractive interactions, ${}_{3}$LJ${}_{1.5}$, we can see this tendency even more clearly, since proteins tend to stay outside of the brush compared to their hydrophilic counterparts. This can be explained by the construction of the proteins themselves; their hydrophobic part lies in the inner part of the protein and therefore is screened by their hydrophilic part, making the interaction with A-beads weaker.

The shape of the proteins for each of the systems is depicted through illustration of their asphericity values in Figure A5. The shape of the hydrophobic A-bead case seems to be remain relatively unaltered as an average. This can be a result of two factors: (i) the hydrophobic interactions within the protein are strong enough and want to keep the protein as spherical as possible and (ii) the hydrophilic parts of the protein have more freedom to move without a big energy cost compared to their hydrophobic counterparts, which grants them more freedom to vary the shape of the protein due to the A-bead attraction.
